# The Importance of Glycerophospholipid Production to the Mutualist Symbiosis of Trypanosomatids

**DOI:** 10.3390/pathogens11010041

**Published:** 2021-12-31

**Authors:** Allan C. de Azevedo-Martins, Kary Ocaña, Wanderley de Souza, Ana Tereza Ribeiro de Vasconcelos, Marta M. G. Teixeira, Erney P. Camargo, João M. P. Alves, Maria Cristina M. Motta

**Affiliations:** 1 Laboratório de Ultraestrutura Celular Hertha Meyer, Instituto de Biofísica Carlos Chagas Filho, Universidade Federal do Rio de Janeiro, Rio de Janeiro 20000-000, RJ, Brazil; allan@biof.ufrj.br (A.C.d.A.-M.); wsouza@biof.ufrj.br (W.d.S.); 2 Laboratório Nacional de Computação Científica, Petropolis 25600-000, RJ, Brazil; karyann@lncc.br (K.O.); atrv@lncc.br (A.T.R.d.V.); 3 Instituto Nacional de Ciência e Tecnologia em Biologia Estrutural e Bioimagens, Rio de Janeiro 20000-000, RJ, Brazil; 4 Departamento de Parasitologia, Instituto de Ciências Biomédicas, Universidade de São Paulo, Sao Paulo 05508-000, SP, Brazil; mmgteix@icb.usp.br (M.M.G.T.); erney@usp.br (E.P.C.)

**Keywords:** glycerophospholipids (GPLs), Symbiont Bearing Trypanosomatids (SHTs), symbiotic relationship, phospholipid metabolism, phylogenetic analysis

## Abstract

The symbiosis in trypanosomatids is a mutualistic relationship characterized by extensive metabolic exchanges between the bacterium and the protozoan. The symbiotic bacterium can complete host essential metabolic pathways, such as those for heme, amino acid, and vitamin production. Experimental assays indicate that the symbiont acquires phospholipids from the host trypanosomatid, especially phosphatidylcholine, which is often present in bacteria that have a close association with eukaryotic cells. In this work, an in-silico study was performed to find genes involved in the glycerophospholipid (GPL) production of Symbiont Harboring Trypanosomatids (SHTs) and their respective bacteria, also extending the search for trypanosomatids that naturally do not have symbionts. Results showed that most genes for GPL synthesis are only present in the SHT. The bacterium has an exclusive sequence related to phosphatidylglycerol production and contains genes for phosphatidic acid production, which may enhance SHT phosphatidic acid production. Phylogenetic data did not indicate gene transfers from the bacterium to the SHT nucleus, proposing that enzymes participating in GPL route have eukaryotic characteristics. Taken together, our data indicate that, differently from other metabolic pathways described so far, the symbiont contributes little to the production of GPLs and acquires most of these molecules from the SHT.

## 1. Introduction

Symbiosis means living together and represents a major driver in evolution. Symbiotic relationships occur between living beings of different species that co-evolve and constitute excellent models to understand the origin of organelles in the eukaryotic cell [1]. Nowadays, it is considered that symbiosis comprehends a full range of interactions, not only the beneficial ones but also neutral relations and even those in which one of the partners is harmed, such as parasitism. All trypanosomatid protozoa are parasites; only some are invertebrates (monoxenics), while others alternate between invertebrates and vertebrates (heteroxenics). Among the monoxenics, seven species pertaining to the *Angomonas*, *Kentomonas,* and *Strigomonas* genera (strigomonads) evolved through a mutualistic relationship with an intracellular bacterium referred to as Trypanosomatid Proteobacterial Endosymbiont (TPE), belonging to the *Candidatus* Kinetoplastibacterium genus [2,3]. This prokaryote is a gram-negative β-proteobacterium from the Alcaligenaceae family, and it is related to *Achromobacter piechaudii*, *Taylorella equigenitalis,* and some species from the Bordetella genus [2,3,4,5]. Another genus of symbiont-harboring trypanosomatids (SHTs) is *Novymonas*. In this case, the β-proteobacterium belongs to the Burkholderiaceae family and is important to the host-cell fitness although a small part of cultures is symbiont-free [6]. Considering that the eukaryotic cell with its structures resulted from the symbiosis between different primitive microorganisms [7], SHTs constitute interesting models to study cell evolution.

The presence of the symbionts in strigomonads is associated with ultrastructural alterations in the host trypanosomatid, such as an atypical microtubule array, a reduced paraflagellar rod, and a looser arrangement of the kinetoplast DNA network [8,9]. Furthermore, the symbiont is also related to modifications on the host surface charge and plasma membrane carbohydrate composition that somehow hampers the interaction and even impairs the insect host colonization by aposymbiotic trypanosomatids. Such strains were obtained after antibiotic treatment and are only maintained in vitro [10,11,12,13]. In the *Angomonas* and *Strigomonas* genera, predictions from genome annotations were consistent with the first biochemical descriptions, showing that the symbiont completes essential biosynthetic routes of the host cell, such as those for heme, amino acid, and vitamin production [10,14,15,16,17,18,19,20]. It means that SHTs present very low nutritional requirements and reduced growth rates when compared to trypanosomatids that naturally do not contain the symbiont and are usually referred to as regular trypanosomatids (RTs).

In fact, the endosymbiosis in trypanosomatids has been characterized by two-way metabolic changes. In this sense, the TPE enhances the mitochondrion’s oxidative phosphorylation and induces an increase in energy production by making the metabolic link between glycosomes and the mitochondrion. The result of the interaction between these three cell structures is the increased production but also the higher consumption of ATP. There are indications that this integrated metabolism benefits the bacterium, which is able to use part of the generated metabolites to build its own carbon skeleton [21,22]. The presence of the TPE also increases host phospholipid production, especially for phosphatidylcholine (PC). Once isolated from the host cell, the bacterium is able to survive for 3 h and to produce phospholipids in media containing [^32^P]-orthophosphate. However, the PC amounts in the free symbiont are markedly reduced when compared to those measured in the intracellular bacterium, thus indicating that this prokaryote may obtain most of its PC from the host trypanosomatid [23].

The glycerophospholipids (GPL) are amphipathic molecules composed by hydrophilic head groups linked via phosphate to glycerol-bound fatty acid or fatty alcohol chains. GPLs are present in both eukaryotic and prokaryotic cells and have structural and metabolic functions. They play essential roles as membrane constituents, regulating protein structure and function as well as the formation of specialized membrane domains [24,25]. Furthermore, GPLs are involved in cell cycle and signaling, including in trypanosomatids [26,27,28,29]. In the *Trypanosoma* and *Leishmania* genera, PC and phosphatidylethanolamine (PE) are present in higher amounts, followed by phosphatidylinositol (PI), while phosphatidylserine (PS), phosphatidylglycerol (PG), and cardiolipin (CL) are present in minor quantities [30,31,32,33,34]. Similarly, in the SHT *Angomonas deanei*, biochemical analysis showed that the major phospholipids are PC and PE, however PI and CL are present in similar quantities that correspond to those found in RTs [35].

The symbiotic bacterium of *A. deanei* presents CL as the major phospholipid, followed by equal amounts of PC and PE and minor quantities of PI [35]. PC is unusual in prokaryotes, which usually contain PG as the main structural phospholipid in membranes, except in those that maintain a close symbiotic or pathogenic relationship with eukaryotic cells [36,37,38,39,40,41]. PI is also involved in symbiotic interactions by regulating intracellular calcium levels in the host in a phosphoinositide-dependent signaling pathway, as reported in Rhizobium-legume symbiosis [42,43]. In *A. deanei*, genome analyses and phylogenetic data showed that sequences for PI biosynthesis were absent in the symbiont, thus indicating that the bacterium depends on the host to obtain PI [44].

In this work, we performed in-silico identification and phylogenetic analysis of the genes involved in glycerophospholipid synthesis considering strigomonad SHTs and their respective symbionts as well as other members of the family that naturally do not contain the symbiotic bacterium. The symbiont but not the host trypanosomatid has the gene that converts phosphatidylglycerol phosphate (PGP) to PG. Furthermore, the bacterium contains genes that may enhance the host production of phosphatidic acid (PA), which is essential for the construction of glycerophospholipids. Such sequences are also present in SHTs and have eukaryotic origin. However, most genes of the metabolic pathways using dihydroxyacetone phosphate (DHAP) and glycerol-3-phosphate (G3P) to produce PS, PE, PG, and CL are complete in the host trypanosomatid genome but absent in the symbiont. Here, our genomic data indicate that the symbiont seems to depend on the host cell for obtaining most glycerophospholipids that constitute its membranes.

## 2. Results

### 2.1. TPE Phylogenies

TPE sequences for G3P dehydrogenase (Figure S1) cluster and share two common ancestors with two *Bordetella* species. Sequences for lysophosphatidate acyltransferase and PA citidyltransferase (Figures S2 and S3), involved in the production of CDP-diacylglycerol from G3P, and the sequences of phosphatidylglycerophosphatase (Figure S4), which produces PG from PGP, present a common ancestor for TPE and bacteria of the *Taylorella* genus. These sequences cluster in a distant branch from the *Achromobacter* and *Bordetella* genera due to the divergence between sequences. In phylogenetic trees for PGP synthase and PS decarboxylase (Figures S5 and S6), the symbiont is positioned as a basal group in the Alcaligenaceae family.

### 2.2. RT and SHT Phylogenies

Most of the trypanosomatid enzymes involved in phospholipid synthesis analyzed here presented no sign of horizontal gene transfer (data not shown), but a few of them have more ambiguous scenarios.

**Figure 1:**
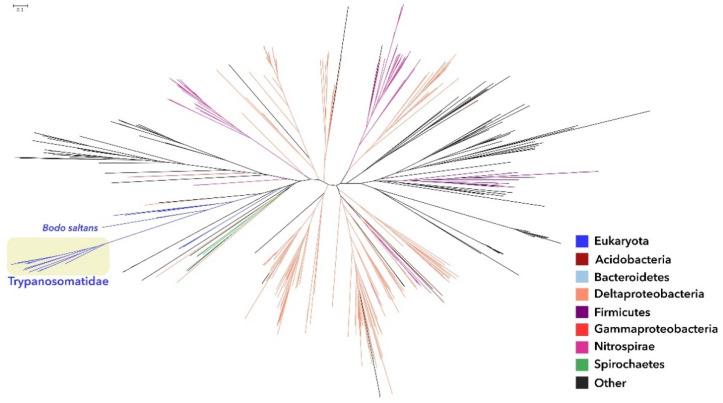
Maximum likelihood phylogenetic tree for enzyme glycerol-3-phosphate dehydrogenase (1.1.1.8), using *L. major* sequence as the query for sequence selection. Branch colors identify taxonomic affiliation as detailed in the legend. Close-up details of the areas containing the trypanosomatid sequences can be seen in Figure S1. Schemes follow the same formatting.

G3P dehydrogenase (1.1.1.8 and 1.1.5.3) was found in many trypanosomatids as well as *Bodo saltans*, plus a few other eukaryotes (Figure 1 and Figure S7 for close-up details). The 1.1.1.8 tree is mainly composed by bacteria (mostly Deltaproteobacteria and Nitrospirae). The other Eukaryota in this tree are all near the Kinetoplastida, including the unexpected grouping of *B. saltans* with the alveolate *Perkinsus marinus*, with bootstrap support value (BSV) of 70. The few other eukaryotes clustering with the trypanosomatids with high BSV (100) are from diverse parts of the tree of eukaryotes: *Vitrella brassicaformis* (Alveolata), *Ectocarpus silicosus* (Phaeophyceae, brown alga), and *Sphaeroforma antarctica* (Ichthyosporea, near animals).

The sister group of these eukaryotes in the tree, with BSV of 86, is composed of one unclassified Proteobacteria and one unclassified Deltaproteobacteria. All other nearby clades in the tree present very low statistical support and therefore do not allow for any confident definition of a more exact placement of this trypanosomatid enzyme in the tree. The only other eukaryotes present in the 1.1.1.8 tree are a group of three green algae; they are relatively near the trypanosomatid clade, but with very low BSV; therefore, their placement is not conclusive.

**Figure 2:**
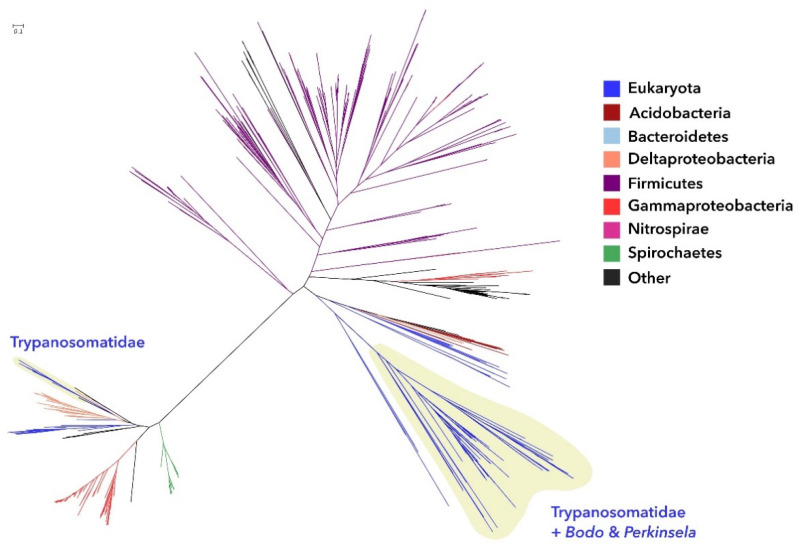
Maximum likelihood phylogenetic tree for enzyme glycerol-3-phosphate oxidase (1.1.3.21), using *L. major* sequence as the query for sequence selection. Branch colors identify taxonomic affiliation as detailed in the legend. Close-up details of the areas containing the trypanosomatid sequences can be seen in Figure S2.

The tree for G3P oxidase (Figure 2 and Figure S8) presents two clades of trypanosomatids: a larger one containing most trypanosomatids, including *Trypanosoma*, *Bodo*, and *Perkinsela*; and a smaller one, comprised of mostly *Leishmania* but also containing one *T. theileri* and a few *Leptomonas*, *Strigomonas*, and *Angomonas* sequences. Since the two clades are located in two different subtrees separated by a long branch, it is possible that the two clades are either different families performing the same function (G3P oxidase) or different but related functions, and hence, the sequence similarity allows for the selection and alignment of both groups. Closer inspection of RPSBLAST search results (against the Conserved Domain Database of NCBI) representative couples, one eukaryotic and one bacterial, from each of these two clades, suggests these are indeed two separate families of 2-hydroxyacid phosphoglycerate dehydrogenases since the domains identified have the same functions but different sequences (data not shown).

It is interesting to notice that the large clade contains two groups of Strigomonadinae sequences, one in their expected placement near the Leishmaniinae and another (composed of just *A. deanei* and *S. culicis* sequences as well as one *Leptomonas pyrrhocoris* sequence) as an outgroup to all other Kinetoplastida sequences, including *B. saltans* and *Perkinsela* sp.

As seen for the G3P dehydrogenase, the G3P oxidase tree is mainly composed of enzymes from bacteria (Firmicutes, Gammaproteobacteria, Spirochaetes, and Deltaproteobacteria), being the most represented groups, but there are more eukaryotic groups present. In the large clade, the Eukaryota are near each other although with very low BSV, which does not allow for confident placement. The sister group of the Kinetoplastida clade in this subtree is comprised of two Ciliates (*Pseudocohnilembus* and *Oxytricha*) and one dinoflagellate (*Symbiodinium*), with BSV of 58. More distantly, and with insignificant statistical support, there is a small group of Apicomplexa, plants, and Stramenopiles intermingled with Bacteria, mostly Deltaproteobacteria and Acidobacteria. The fourth and final clade containing Eukaryota is in the smaller subtree, and it does not cluster close to the trypanosomatids in that subtree.

**Figure 3:**
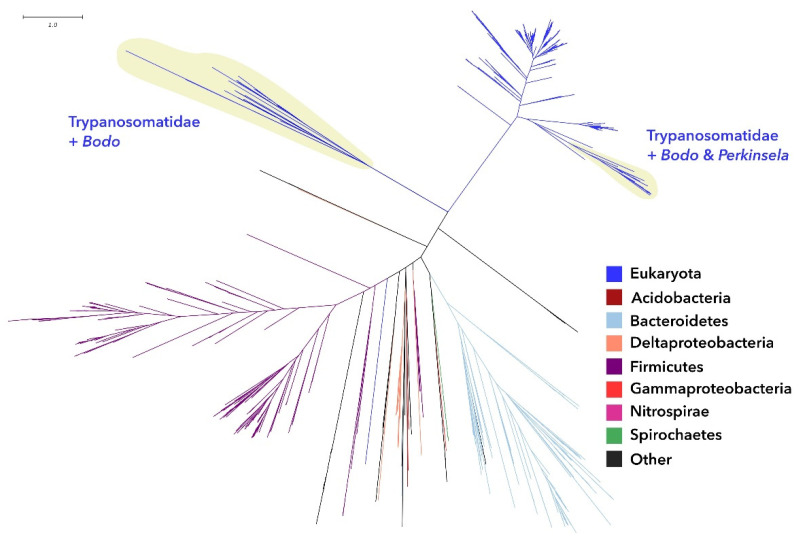
Maximum likelihood phylogenetic tree for enzyme phosphatidic acid cytidylyltransferase (2.7.7.41), using *L. major* sequence as the query for sequence selection. Branch colors identify taxonomic affiliation as detailed in the legend. Close-up details of the areas containing the trypanosomatid sequences can be seen in Figure S3.

The PA cytidylyltransferase tree (Figure 3 and Figure S9) presents a similar situation to that for enzyme G3P oxidase, whose tree is divided in two subtrees separated by a long branch, indicating that at least two different gene families are involved. One of the subtrees is comprised of Eukaryota and contains a diverse group of Kinetoplastida, including *Bodo* and *Perkinsela* as well as many Trypanosomatidae genera. The other subtree contains almost only Bacteria, mainly Firmicutes and Bacteroidetes, but also a large group of Trypanosomatidae. However, this clade is better said to be in-between the two subtrees and does not cluster with good statistical support with any bacterial group. Therefore, it is not possible to conclude whether this is a very different variant of PA cytidylyltransferase that is exclusive to trypanosomatids or the result of an ancient horizontal gene transfer.

## 3. Discussion

Symbiotic relationships constitute excellent models for the study of cell evolution, such as the origin of organelles. In symbioses that have been investigated so far, it is common to observe a modulation of lipid metabolism on both partners. In the association of sea anemones and corals with *Symbiodinium*, the dinoflagellate influences the fatty acid composition of the host lipid bodies [45,46,47,48,49,50,51,52,53,54]. There are also reports showing the influence of symbionts in the host-cell membrane phospholipids in beneficial or pathogenic associations. For example, in relationships between prokaryotes and eukaryotes, PC, a GPL usually absent in prokaryotes, is present in the membrane of *Pseudomonas aeruginosa*, *Brucella abortus,* and *Agrobacterium tumefaciens* [55,56,57]. Another case is the symbiotic relationship between leguminous plants and the symbiotic bacterium *Rhizobium leguminosarum*, where there is an increase in PI 4-phosphate content in the host plant, which is necessary for the root nodulation process to occur [58]. Still regarding GPLs, some intracellular pathogens, such as those from the *Yersinia* genus, have mechanisms to subvert the phosphoinositide metabolism, altering the amounts of PI, PIP, and PIP2 in the host in order to promote or to block their internalization in cells of different tissues [59].

**Figure 4:**
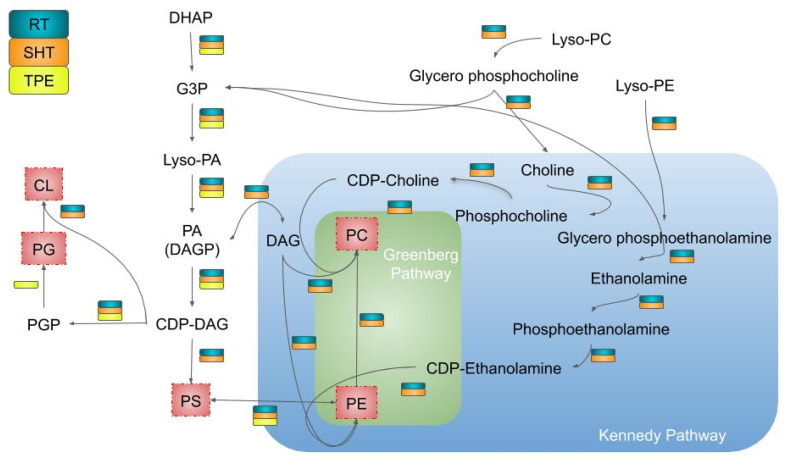
Glycerophospholipid (GPL) metabolism in regular trypanosomatids (RT), symbiont harboring trypanosomatid (SHT), and trypanosomatid proteobacterial endosymbiont (TPE). The scheme summarizes the metabolic pathways of GPL synthesis considering the genes identified in RT, SHT, and *Candidatus* Kinetoplastibacterium crithidii (*A. deanei* symbiont). The synthesis of GPL can use metabolic intermediates, such as DHAP and G3P, for the synthesis of PA, a central molecule in the metabolism of GPL. In RTs and SHTs, CDP-DAG is produced from PA, thus enabling the synthesis of zwiterionic GPL (PS, PE, and PC) or the production of PGP. It is important to note that in these organisms, the synthesis of CL does not pass through PGP but occurs through the metabolites CDP-DAG and PG to form CL. Differently, the TPE can use PGP to produce PG, but genes encoding prokaryotic enzymes that produce CL have not been found. RT and SHT present genes for PC synthesis from PE methylation through the Greenberg pathways (highlighted in the green background) and through de-novo synthesis via the Kennedy pathway (highlighted in the blue background). PE can be produced from the Kennedy pathway or in reverse from PS (the latter path was also identified in the symbiont). One possibility is that a phospholipase D (PLD) could act on the metabolism of lyso-PC and lyso-PE molecules, which could be obtained from the environment and used as a source to produce GPL. For detailed identification of enzymes and molecules of these pathways, check Table S1 and Figure S10. CL, cardiolipin; DAG, diacylglycerol; DAGP, diacylglycerol phosphate; DHAP, dihydroxyacetone; G3P, glycerol 3 phosphate; Lyso-PA, lyso-phosphatidic acid; Lyso-PC:, lso-phosphatidylcholine; Lyso-PE, lyso-phosphatidylethanolamine; PA, phosphatidic acid; PC, phosphatidylcholine; PE, phosphatidylethanolamine; PG, phosphatidylglycerol; PGP, phosphatidylglycerolphosphate; PS, phosphatidylserine; RT, regular trypanosomatids; SHT, symbiont harboring trypanosomatids; TPE, trypanosomatid proteobacterial endosymbiont.

Although the amount of GPLs varies in eukaryotes and prokaryotes, their membranes have a very similar biochemical structure, consisting of G3P, a phosphorylated alcohol, linked to two acyl chains [60]. This results in PA production, a molecule that is initially synthesized in the ER membrane of eukaryotes and represents an important component in establishing the mutualistic relationship between *Rhizopus microsporus* fungi and *Burkholderia* endobacteria [61]. In trypanosomatids, such as *A. deanei* and other strigomonad SHTs analyzed in this work, sequences for enzymes that use DHAP for PA synthesis are present in the genome. This pathway that contains three chemical reactions is catalyzed in the first step by one enzyme (a G3P dehydrogenase (1.1.1.8 or 1.1.5.) or G3P oxidase (1.1.3.21)) to produce G3P, followed by addiction of acyl chains to G3P molecule by G3P acyltransferase to produce PA (Figure 4 and Figure S10).

In strigomonad trypanosomatids, the symbiont has the gene for G3P dehydrogenase enzyme (1.1.1.94), which uses DHAP for G3P synthesis, indicating the relevance of G3P to the bacterium and corroborating data on the ability of SHTs to ferment. Such protozoans prioritize carbohydrate consumption in glucose-enriched media, producing large amounts of G3P when oxidative phosphorylation is inhibited by KCN [22]. In the symbiosis between fungi and bacteria, G3P is involved not only in energy metabolism but especially in GPL biosynthesis, which is regulated by the Hog1 MAPK pathway [61]. Considering the course of evolution, this fermentative activity represents a possibility of an alternative energy supply in relation to oxidative phosphorylation since trypanosomatids diverged very early from the last eukaryotic common ancestor [62].

PA production also seems to be possible in symbiotic SHT bacteria since the gene of a G3P acyltransferase (2.3.1.51), which converts Lyso-PA to PA, is present. As stated earlier, in corals, the *Symbiodinium* endosymbiont has the ability to influence the composition of PA fatty acids found in lipid bodies and other host structures [63]. It is noteworthy that PA can be dephosphorylated into diacylglycerol (DAG) by enzyme PA phosphatase (3.1.3.4) and vice versa by enzyme DAG kinase (2.7.1.107). Genes for both enzymes PA phosphatase and DAG kinase were identified in endosymbionts and in host trypanosomatids (Figure 4 and Figure S10). These two molecules, PA and DAG, represent starting points from which the GPL metabolic pathways branch out, resulting in the production of different types of phospholipids [60,64].

DAG is used to produce PC and PE by the Kennedy pathway through a condensation reaction with the radical, choline, or ethanolamine, respectively. An assumption is that this pathway, present in the ER, uses exogenous Lyso-PE or Lyso-PC so that the trypanosomatid could obtain ethanolamine or choline from the external environment. Genes for enzymes involved in PE and PC production showed high similarities between protozoa species, such as *T. cruzi* and *Plasmodium falciparum* [65,66]. Genes for the Kennedy pathway were not identified in strigomonad TPEs (Figure 4).

The Greenberg pathway is also present in trypanosomatids; in this case, three successive PE methylations generate PC, the major GPL of eukaryotic cell membranes. Although it is known that in eukaryotes, more than one PE N-methyltransferase (2.1.1.17) participates in this route, we have identified only one gene for this enzyme in *A. deanei*. It is possible that this single enzyme is catalyzing the three methylations, thus producing PC, as described in *L. major*, which contains the LmjPEM2 gene [67]. In strigomonad TPEs, as expected for prokaryotes, we did not identify genes for this pathway (Figure 4).

**Figure 5:**
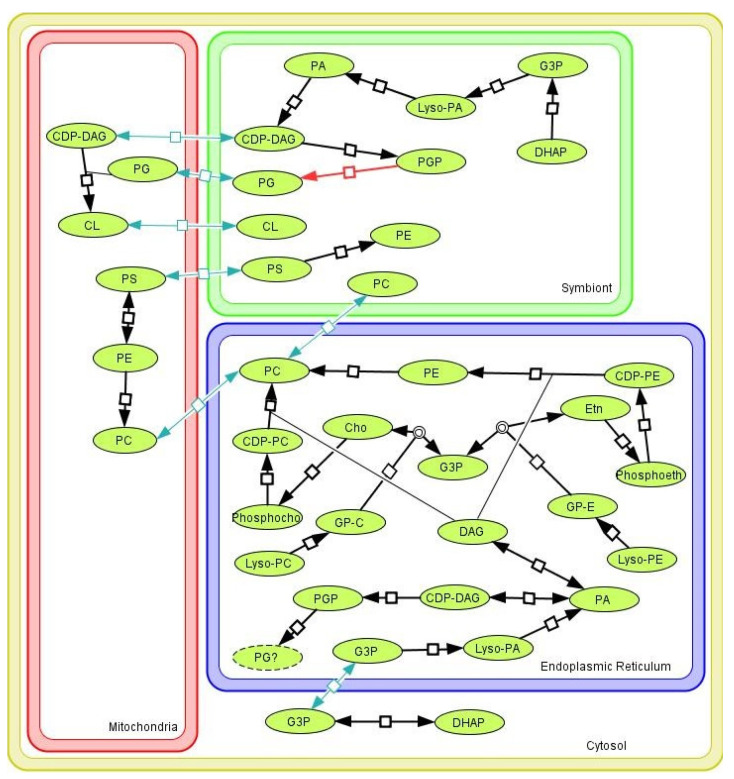
Glycerophospholipid (GPL) metabolism pathways in symbiont harboring trypanosomatids (SHTs) considering different cell compartments. The figure presents the reactions of GPL metabolism localized in the cytosol (yellow compartment), endoplasmic reticulum (blue compartment), mitochondrion (red compartment), and the symbiont (trypanosomatid proteobacterial endosymbiont; TPE, green compartment). Black arrows indicate enzyme reactions identified in genome searches. Regarding PG formation in the SHT endoplasmic reticulum (represented with dotted line), the enzyme that catalyzes its synthesis from PGP in the final step was found neither in SHT nor in regular trypanosomatids (RT) genomes but in the symbiont (red arrow). It is possible that the PG produced in the TPE could be exported to the mitochondrion to enhance CL synthesis in this organelle. Then, CL could be imported by the TPE, which is unable to produce this GPL. In addition, TPEs present a reversible reaction to produce PS from PE, which would regulate both GPL amounts. Green arrows suggest how compartments share GPL molecules. For detailed identification of enzymes and molecules of these pathways, check Table S1. CL, cardiolipin; CDP-DAG, CDP-diacylglycerol; CDP-PC, CDP-phosphatidylcholine; CDP-PE, CDP-phosphatidylethanolamine; DAG, diacylglycerol; DHAP, dihydroxyacetone; G3P, glycerol 3 phosphate; GP-C, glycerophosphocholine; GP-E, glycerophosphoethanolamine; Lyso-PA, lyso-phosphatidic acid; Lyso-PC, lyso-phosphatidylcholine; Lyso-PE, lyso-phosphatidylethanolamine; PC, phosphatidylcholine; PE, phosphatidylethanolamine; PG, phosphatidylglycerol; PGP, phosphatidylglycerolphosphate; PS, phosphatidylserine; Phosphocho, phosphocholine; Phosphoeth, phosphoethanolamine; RT, regular trypanosomatids; SHT, symbiont harboring trypanosomatids; TPE, trypanosomatid proteobacterial endosymbiont.

In addition to DAG, PA is also a starting point for GPL production. The first step is the conversion of PA to CDP-DAG by the enzyme PA cytidylyltransferase (2.7.7.41). From there, CDP-DAG can be used to produce PS by the enzyme CDP-DAG-serine phosphatidyltransferase (2.7.8.8), whose gene has been identified in the trypanosomatids and symbionts analyzed in this work. The presence of this sequence suggests that the PS production pathway occurs in the protozoan ER. In addition, we have also identified the enzymes PS synthase 2 (2.7.8.29) and PS decarboxylase (4.1.1.65) genes, whose activities result in the conversion of PS to PE and vice versa, thus regulating the cellular levels of such GPLs. Interestingly, the enzyme PS decarboxylase (4.1.1.65) is located in mitochondria, whereas enzymes CDP-DAG-serine phosphatidyltransferase (2.7.8.8) and PS synthase 2 (2.7.8.29) are located in the ER (Figure 5). It has been reported that contact regions between ER and mitochondria, referred to as ER-Mitochondria Encounter Structure (ERMES), permit the regulation of PS and PE content in ER [68]. The proximity between these organelles and the symbiont suggests that the bacterium may play a mitochondrial-like role in the recycling of PS into PE for its own use or even in the supply to the host trypanosomatids (Figure 5).

Another important point to discuss concerns the possible influence of the symbiont on CL production, a GPL that confers high permeability selectivity in membranes. In eukaryotes, CL is present in great amounts in the mitochondrial inner membrane, and its biosynthesis is the result of a biochemical reaction promoted by phosphatidyltransferase, also called as cardiolipin synthase (CLS), a phosphatidyltransferase that condenses PG with the Lyso-PA grouping of a CDP-DAG molecule [69]. In prokaryotes, CL plays an important role in forming an ion barrier and establishing a stable membrane domain for the insertion of respiratory complexes [70,71]. In this case, CL synthesis occurs from the condensation of two PGs by the action of a D-like phospholipase [69]. Searches in SHT genomes indicate the presence of genes for the enzyme CDP-DAG-G3P phosphatidyltransferase (2.7.8.5, which uses CDP-DAG to produce PGP) and CLS (uses PG to produce CL). However, the gene for the enzyme phosphatidylglycerophosphatase (3.1.3.27) was not identified. This enzyme is in an intermediate step in the conversion of PGP into PG and was only found in the symbiont (Figure 4 and Figure S10). Taken together, these findings suggest that the host protozoan would be able to produce CL without necessarily producing the PG precursor but also indicate that the symbiotic bacteria could provide part of the required PG that can be used by the host to produce CL, an essential component of the mitochondrial inner membrane. It is worth mentioning that the presence of the symbiotic bacteria increases the phosphorylative capacity of the host cell [21,22].

Phylogenetic analyses revealed that most of the trypanosomatid enzymes involved in phospholipid synthesis analyzed here presented no sign of horizontal gene transfer, but a few of them have more ambiguous scenarios. The enzyme G3P dehydrogenase (1.1.1.8) was found in trypanosomatids, in *Bodo saltans,* and few other eukaryotic species. The observation that eukaryotes present in the G3P dehydrogenase tree are clustering near each other and, without any sign of artifacts, such as long-branch attraction, suggests that they inherited the enzyme from a common ancestor, while all other eukaryotes lost this particular orthologue. It is not clear why so few and so distantly related groups would retain this version of the enzyme. Another interesting point was the phylogenetic analysis for enzymes G3P oxidase (1.1.3.21) and PA cytidylyltransferase (2.7.7.41) since they grouped trypanosomatids in two clades that are located in two different subtrees separated by a long branch. It is possible that the two clades are either different families performing the same enzyme function or distinct but related functions. Phylogenetic analyses showed that enzymes involved in GPL synthesis positioned symbionts of trypanosomatids as a basal group in the Alcaligenaceae family. Sequences for CDP-DAG and PG production showed a common ancestor for TPEs and bacteria of the *Taylorella* genus, which is in accordance to previous evolutionary data using genomic and phylogenomic analysis [4].

Endosymbionts of trypanosomatids have a very small but highly functional genome [20]. Previous studies showed that these bacteria contain most genes to produce heme, amino acids, and vitamins, thus completing SHT essential metabolic pathways [17,18,19]. In addition, the presence of prokaryotes modulates the expression of host genes, especially those involved in energy metabolism, an important aspect for understanding the co-evolution in SHTs [72,73]. In this work, we found that the symbiont contains few genes to produce GPLs; however, those that are present contribute to the production of phospholipids, such as PG and CL, that seem relevant for the maintenance of symbiont–trypanosomatid mutualistic relationship. The absence of bacterial genes for PC production in the symbiont reinforces the relevance of this GPL in the process of interaction between prokaryotes and eukaryotes; thus, the host could control bacterial growth and division [40].

In-silico data generated here reinforce the idea previously obtained through biochemical analysis that the bacterium GPLs are mainly obtained from the host protozoan. Such results are relevant since they suggest that this phenomenon occurs in different species of strigomonads. More recently, it was shown in *A. deanei* that gene expression can be modulated by RNA interference and that the CRISPRCas9 gene deletion system is active in this protozoan [74,75]. Thus, the present study is very promising for future investigations, indicating how interfere in glycerophospholipid synthesis pathways and revealing the contribution of these molecules to the symbiotic relationship maintenance.

## 4. Materials and Methods

### 4.1. Genomic Analysis

For genomic analysis, protein sequences involved in GPL synthesis were identified by Enzyme Commission (EC) number (Table S1), collecting candidate orthologs from several organisms considering strigomonad SHTs and their respective TPEs. In order to ensure that *A. deanei* and its symbiont, named *Ca*. K. crithidii, presented the target genes, we first searched the sequences by EC number in *L. major* in the TriTrypDB database [45] and *Escherichia coli* in NCBI’s non-redundant (nr) protein database. Then, we searched the *A. deanei* and *Ca*. K. crithidii genomes for these sequences. and each sequence identified was used to create one dataset.

The strigomonad SHT and RT datasets, identified by EC number, are the results of BLASTP searches against the NCBI’s nr database, collecting up to five hundred (for less conserved sequences) to a thousand sequences (for more conserved ones) that passed the maximum threshold E-value of 1 × 10^−10^.

### 4.2. Phylogenetic Analysis

For TPEs, 18 GPL enzyme sequence datasets were created using Betaproteobacteria as reference: six were found in at least two TPEs and were used for phylogenetic analysis, whereas the other 12 sequences could not be used for this purpose (Table 1). For trypanosomatids, 21 datasets were created (Table 2).

For each bacterial dataset, multiple sequence alignments (MSA) were performed with Mafft v.7.313 [46], and evolutionary models were obtained with ModelGenerator [47] (see Table 1 and Table 2). Maximum likelihood phylogenetic inferences were performed by RAxML v.8.2.9 [48] on a Linux computer (BullX B700) cluster using five nodes, each one carrying two Intel Xeon E5-2695v2 Ivy Bridge (24-core) processors, totaling 120 tasks processed by the MPI version of RAxML. One hundred different best tree searches were performed, and the tree with best likelihood found was kept. RAxML’s rapid bootstrap was performed with 100 pseudoreplicates, and support is only shown for branches with values of at least 50. The final tree was drawn and basically formatted by MEGA 7.0.21 [49].

For the eukaryotic datasets, protein alignments were performed using MUSCLE v. 3.8.31 [50], and phylogenetic analyses were performed using RAxML, using gamma-distributed heterogeneity of substitution rates and automatic empirical model selection. Number of bootstrap pseudoreplicates was also automatically selected by the program, ensuring that at least 100 pseudoreplicates were performed. Trypanosomatid trees were drawn and edited in Dendroscope [51], and final cosmetic adjustments were performed with Inkscape (https://inkscape.org/, accessed on 14 October 2021).

## Figures and Tables

**Table 1:** GPL enzyme gene sequences found in the TPEs used for phylogenetic analysis.

E.C.	TPE	Accession	Evolutionary Model
1.1.1.94	*Ca.* K. blastocrithidii	WP_015390063.1	WAG + G
	*Ca.* K. crithidii	WP_015238735.1	
	*Ca.* K. desouzaii	WP_015396598.1	
	*Ca.* K. oncopeltii	WP_015397299.1	
2.3.1.51	*Ca.* K. blastocrithidii	WP_015390063.1	WAG + G + F
	*Ca.* K. crithidii	WP_015389151.1	
	*Ca.* K. desouzaii	WP_015396525.1	
	*Ca.* K. galatii	WP_015389750.1	
	*Ca.* K. oncopeltii	WP_015397233.1	
2.7.7.41	*Ca.* K. crithidii	WP_015389094.1	WAG + I + G + F
	*Ca.* K. desouzaii	WP_015396290.1	
	*Ca.* K. galatii	WP_015389515.1	
	*Ca.* K. oncopeltii	WP_015397011.1	
	*Ca.* K. sorsogonicusi	AWD32429.1	
2.7.8.5	*Ca.* K. blastocrithidii	WP_015238268.1	CPREV + G + F
	*Ca.* K. crithidii	WP_015389066.1	
	*Ca.* K. desouzaii	WP_015396149.1	
	*Ca.* K. galatii	WP_015389380.1	
	*Ca.* K. oncopeltii	WP_015396882.1	
	*Ca.* K. sorsogonicusi	AWD32310.1	
3.1.3.27.A	*Ca.* K. blastocrithidii	WP_015237959.1	CPREV + G + F
	*Ca.* K. crithidii	WP_015238600.1	
	*Ca.* K. desouzaii	WP_015396243.	
	*Ca.* K. galatii	WP_015389467.1	
	*Ca.* K. oncopeltii	WP_015396965.1	
4.1.1.65	*Ca.* K. crithidii	WP_015238529.	WAG + G
	*Ca.* K. desouzaii	WP_015396314.	
	*Ca.* K. sorsogonicusi	AWD32452.1	

G, modeling of heterogeneity of substitution rates using estimates following a discrete gamma distribution; F, fixed residue frequencies; CREPV and WAG, empirical amino acid substitution models as implemented in RAxML (see program documentation for details); 1.1.1.94, Glycerol-3-Phospahte dehydrogenase; 2.3.1.51, Glycerol-3-Phospahte acyltransferase; 2.7.7.41, Phosphatidic Acid cytidylyltransferase; 2.7.8.5, CDP-diacylglycerol- Glycerol-3-Phospahte phosphatidyltransferase; 3.1.3.27.A, phosphatidylglycerophosphatase A; 4.1.1.65, Phosphatidylserine decarboxylase.

**Table 2:** GPL enzyme gene sequences found in *A. deanei* and *S. culicis* used for phylogenetic analysis.

Enzyme	*A. deanei* Accession	*S. culicis* Accession	Substitution Model
1.1.1.8	EPY26396.1	EPY26225.1	LG + G + F
2.3.1.51	EPY39144.1	EPY35625.1	JTT + G + E
3.1.1.5	EPY26987.1	EPY27559.1	LG + G + F
2.7.7.41	EPY27658.1	EPY27813.1	VT + G + E
2.7.8.5	EPY38995.1	EPY20239.1	VT + G + E
4.1.1.65	EPY37752.1	EPY26555.1	JTT + G + E
1.1.3.21	EPY42326.1	EPY33132.1	WAG + G + F
1.1.5.3	EPY29427.1	EPY28316.1	WAG + G + E
2.1.1.17	EPY36199.1	EPY25191.1	LG + G + E
2.3.1.15	EPY32191.1	EPY19897.1	VT + G + F
2.7.1.107	EPY19511.1	EPY28592.1	JTT + G + F
2.7.1.32	EPY36410.1	EPY24705.1	VT + G + F
2.7.1.82	EPY28885.1	EPY29571.1	JTT + G + E
2.7.7.14	EPY43438.1	EPY23528.1	VT + G + F
2.7.7.15	EPY32479.1	EPY36580.1	VT + G + F
2.7.8.1	EPY25282.1	EPY36363.1	JTT + G + E
2.7.8.29	EPY26426.1	EPY26341.1	VT + G + F
2.7.8.2	EPY27385.1	EPY22867.1	VT + G + E
2.7.8.8	EPY26426.1	EPY26341.1	VT + G + F
3.1.3.4	EPY21603.1	Not found	VT + G + E
3.1.4.46	EPY26374.1	EPY27334.1	JTT + G + E

G, modeling of heterogeneity of substitution rates using estimates following a discrete gamma distribution; F, fixed residue frequencies; E, empirical residue frequencies; LG, JTT, VT, and WAG, empirical amino acid substitution models as implemented in RAxML (see program documentation for details); 1.1.1.8, Glycerol-3-Phospahte dehydrogenase; 1.1.3.21, Glycerol-3-Phospahte oxidase; 1.1.5.3, Glycerol-3-Phospahte dehydrogenase; 2.1.1.17, Phosphatidylethanolamine N-methyltransferase; 2.3.1.15, Glycerol-3-Phospahte acyltransferase; 2.3.1.51, Glycerol-3-Phospahte acyltransferase; 2.7.1.32, choline kinase; 2.7.1.82, ethanolamine kinase; 2.7.1.107, diacylglycerol kinase; 2.7.7.14, ethanolamine-phosphate cytidylyltransferase; 2.7.7.15, choline-phosphate cytidylyltransferase; 2.7.7.41, Phosphatidic Acid cytidylyltransferase; 2.7.8.1, ethanolaminephosphotransferase; 2.7.8.2, diacylglycerol cholinephosphotransferase; 2.7.8.29, phosphatidylserine synthase 2; 2.7.8.5, CDP-diacylglycerol- Glycerol-3-Phospahte phosphatidyltransferase; 2.7.8.8, CDP-diacylglycerol-serine phosphatidyltransferase; 3.1.1.5, lysophospholipase I; 3.1.3.4, Phosphatidic Acid phosphatase; 3.1.4.46, glycerophosphodiester phosphodiesterase; 4.1.1.65, Phosphatidylserine decarboxylase.

## Supplementary Materials

The following are available online at https://www.mdpi.com/article/10.3390/pathogens11010041/s1, Table S1: Enzyme list; Figure S1: Maximum likelihood phylogenetic tree for enzymes Glycerol-3-Phospahte dehydrogenase (1.1.1.94) using *E. coli* sequence as the query for sequence selection; Figure S2: Maximum likelihood phylogenetic tree for enzymes Glycerol-3-Phospahte acyltransferase (2.3.1.51) using *E. coli* sequence as the query for sequence selection; Figure S3: Maximum likelihood phylog enetic tree for enzymes Phosphatidic Acid cytidylyltransferase (2.7.7.41) using *E. coli* sequence as the query for sequence selection; Figure S4: Maximum likelihood phylogenetic tree for enzymes CDP-DAG- Glycerol-3-Phospahte phosphatidyltransferase (2.7.8.5) using *E. coli*’s sequence as the query for sequence selection; Figure S5: Maximum likelihood phylogenetic tree for enzymes phosphatidylglycerophosphatase A (3.1.3.27_A) using *E. coli* sequence as the query for sequence selection; Figure S6: Maximum likelihood phylogenetic tree for enzymes Phosphatidylserine decarboxylase (4.1.1.65), using *E. coli*’s sequence as the query for sequence selection; Figure S7: Close-up details maximum-likelihood phylogenetic tree of enzyme Glycerol-3-Phospahte dehydrogenase (1.1.1.8), focusing on the clades containing trypanosomatid sequences; Figure S8: Close-up details maximum-likelihood phylogenetic tree of enzyme Glycerol-3-Phospahte oxidase (1.1.3.21), focusing on the clades containing trypanosomatid sequences; Figure S9: Close-up details maximum-likelihood phylogenetic tree of enzyme Phosphatidic Acid cytidylyltransferase (2.7.7.41), focusing on the clades containing trypanosomatid sequences; Figure S10: Glycerophospholipid (GPL) metabolism in regular trypanosomatids (RT), symbiont harboring trypanosomatid (SHT), and trypanosomatid proteobacterial endosymbiont (TPE).
