# Supplementary material for: The Importance of Glycerophospholipid Production to the Mutualist Symbiosis of Trypanosomatids
Source: Pathogens. 2021 Dec 31;11(1):41. doi: 10.3390/pathogens11010041 (PMC8779180; doi:10.3390/pathogens11010041)
Supplement: Supplementary file 1 [file pathogens-11-00041-s001.zip › S1Table.pdf]

S1 Table. Enzyme list.

E.C.	Description	Reactants	Products
1.1.1.8	G3P dehydrogenase (NAD+)	DHAP	G3P
1.1.1.94	G3P dehydrogenase (NADP+)	DHAP	G3P
1.1.3.21	G3P oxidase	DHAP	G3P
1.1.5.3	G3P dehydrogenase	DHAP	G3P
2.1.1.17	PE methyltransferase	PE + 3(SAM)	PC + 3(SAH)
2.3.1.15	G3Pacyltransferase	Acyl-CoA + G3P	Lyso-PA + CoA
2.3.1.51	Lyso-PA acyltransferase	Acyl-CoA + Lyso-PA	PA + CoA
2.7.1.107	DAG kinase	DAG + ATP	PA + ADP
2.7.1.32	ethanolamine kinase	ethanolamine + ATP	EP + ADP
2.7.1.82	choline kinase	choline + ATP	CP + ADP
2.7.7.41	PA citidilyltransferase	CTP + PA	CDP-DAG + PPi
2.7.7.14	ethanolamine-phosphate cytidylyltransferase	CTP + EP	CDP-ethanolamine + PPi
2.7.7.15	choline-phosphate cytidylyltransferase	CTP + CP	CDP-choline + PPi
2.7.8.1	Ethanolaminephosphotransfe-rase	CDP-ethanolamine + DAG	CMP + PE
2.7.8.2	cholinephosphotransferase	CDP-choline + DAG	CMP + PC
2.7.8.29	PS synthase 2	PE + serine	PS + ethanolamine
2.7.8.41	CL synthase	CDP-DAG + PG	CMP + CL
2.7.8.5	PGP synthase	CDP-DAG + G3P	CMP + PGP
2.7.8.8	PS synthase	CDP-DAG + serine	CMP + PS
3.1.1.5	lysophospholipase	Lyso-PC/Lyso-PE	GP-C/GP-E + carboxylate
3.1.3.4	PA phosphatase	DAGP	DAG + P
3.1.4.46	glycerophosphodiester phosphodiesterase	glycerophosphodiester	G3P
3.1.3.27.A	Phosphatidylglycerophospha-tase A	PGP	PG + P
4.1.1.65	PS decarboxylase	PS	PE + CO2
