# Supplementary figures and images for: The Importance of Glycerophospholipid Production to the Mutualist Symbiosis of Trypanosomatids
Source: Pathogens. 2021 Dec 31;11(1):41. doi: 10.3390/pathogens11010041 (PMC8779180; doi:10.3390/pathogens11010041)

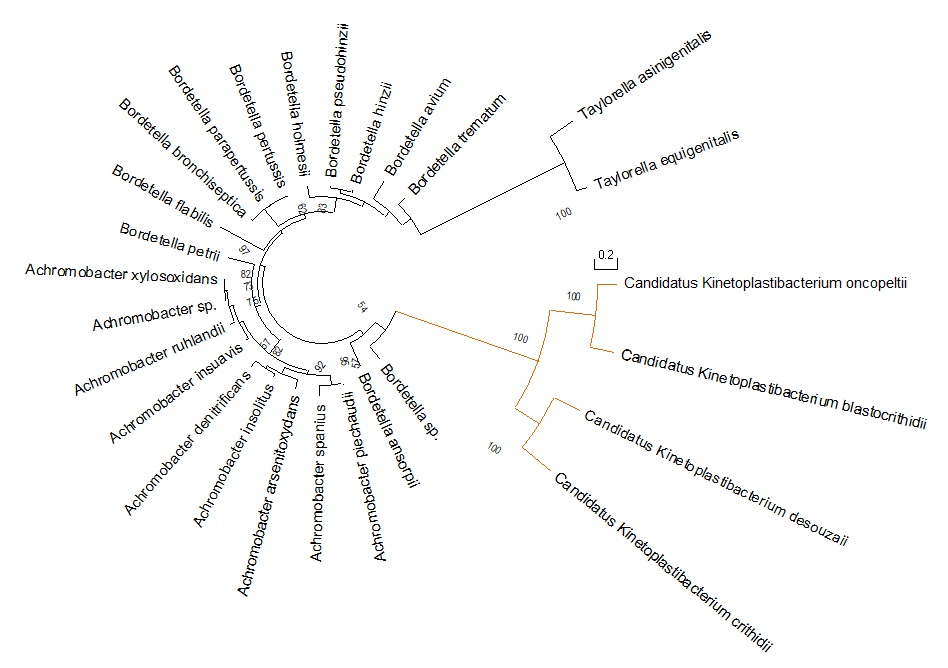

Supplement: Supplementary file 1 [file pathogens-11-00041-s001.zip › SupplFig1.jpg]

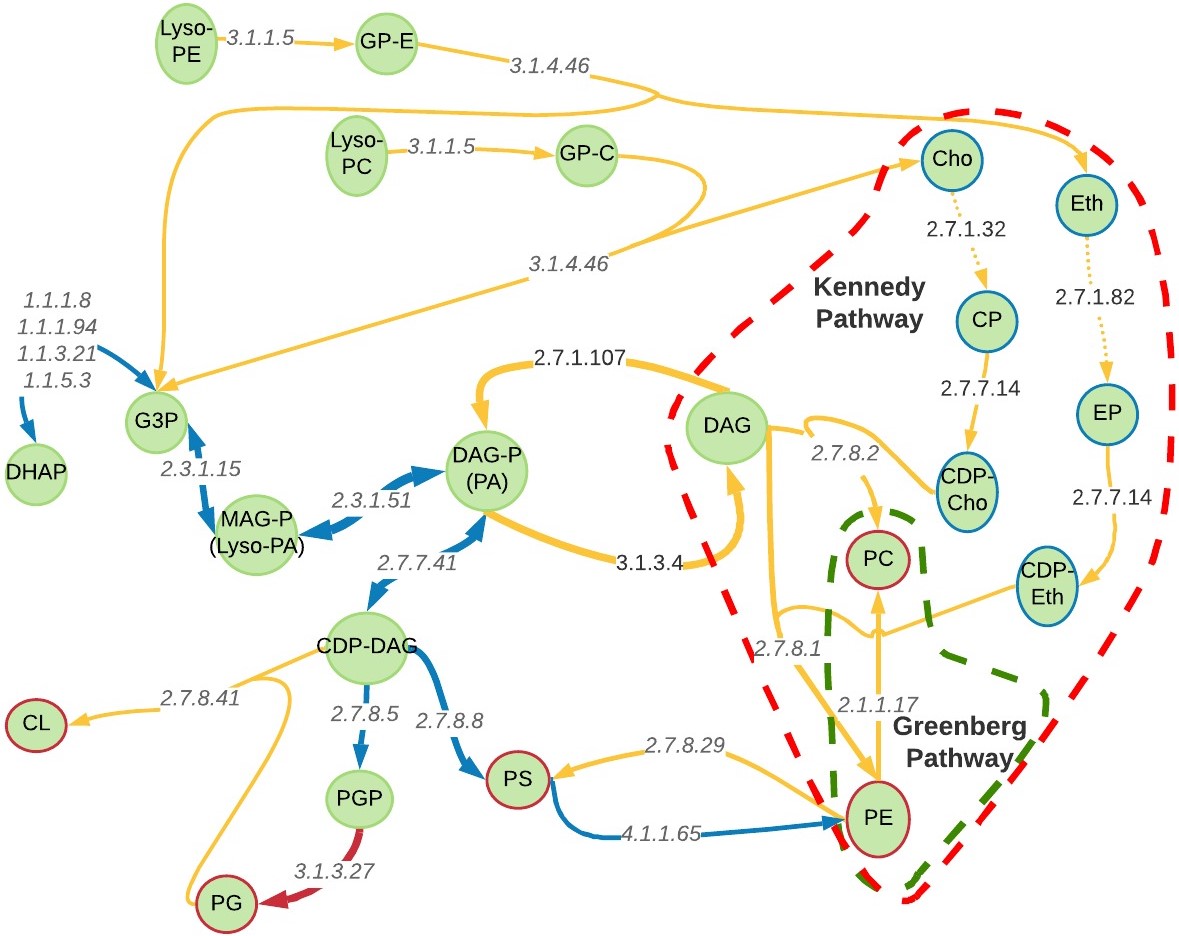

Supplement: Supplementary file 1 [file pathogens-11-00041-s001.zip › SupplFig10.jpeg]

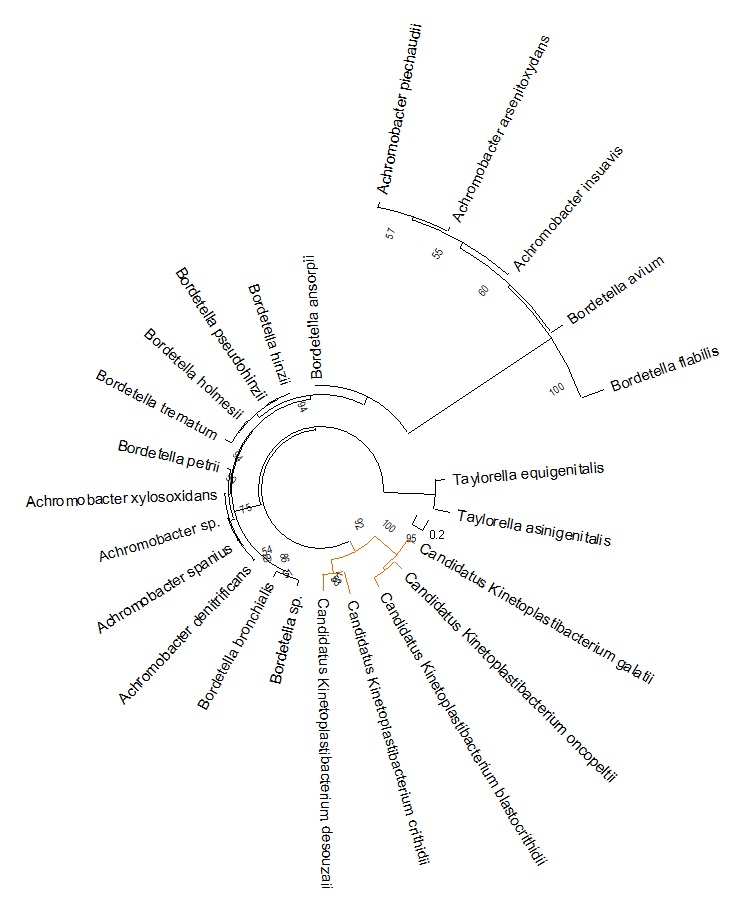

Supplement: Supplementary file 1 [file pathogens-11-00041-s001.zip › SupplFig2.jpg]

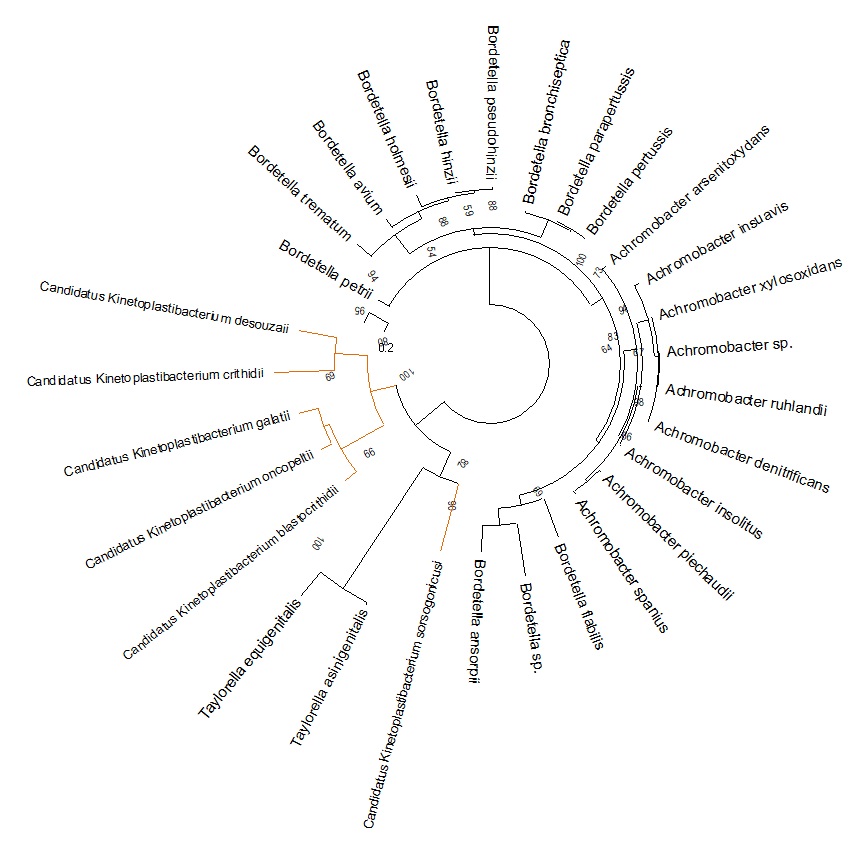

Supplement: Supplementary file 1 [file pathogens-11-00041-s001.zip › SupplFig3.jpg]

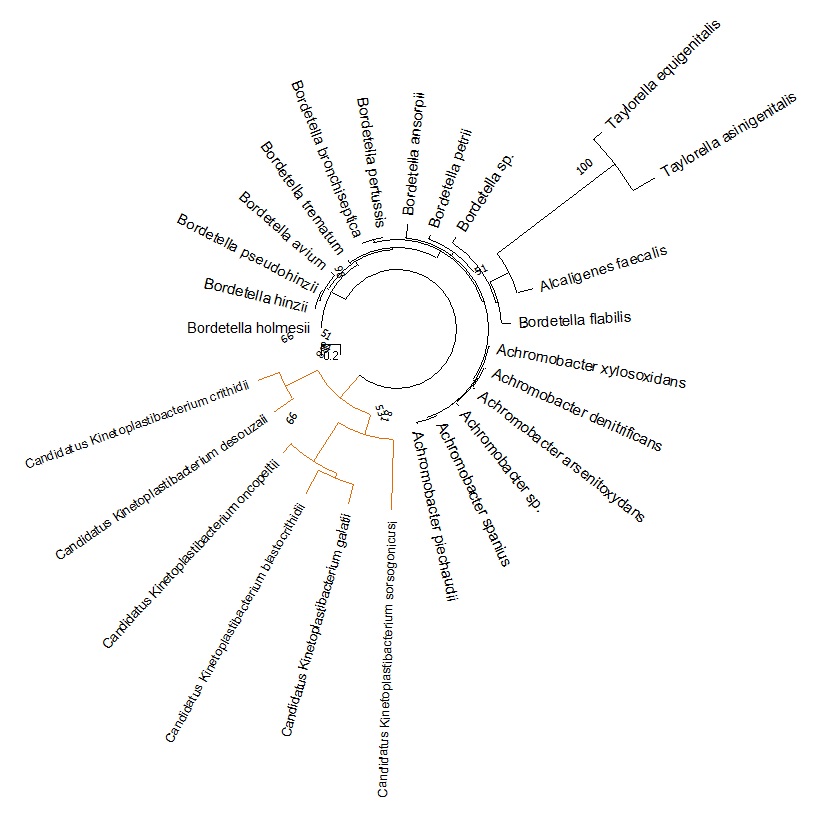

Supplement: Supplementary file 1 [file pathogens-11-00041-s001.zip › SupplFig4.jpg]

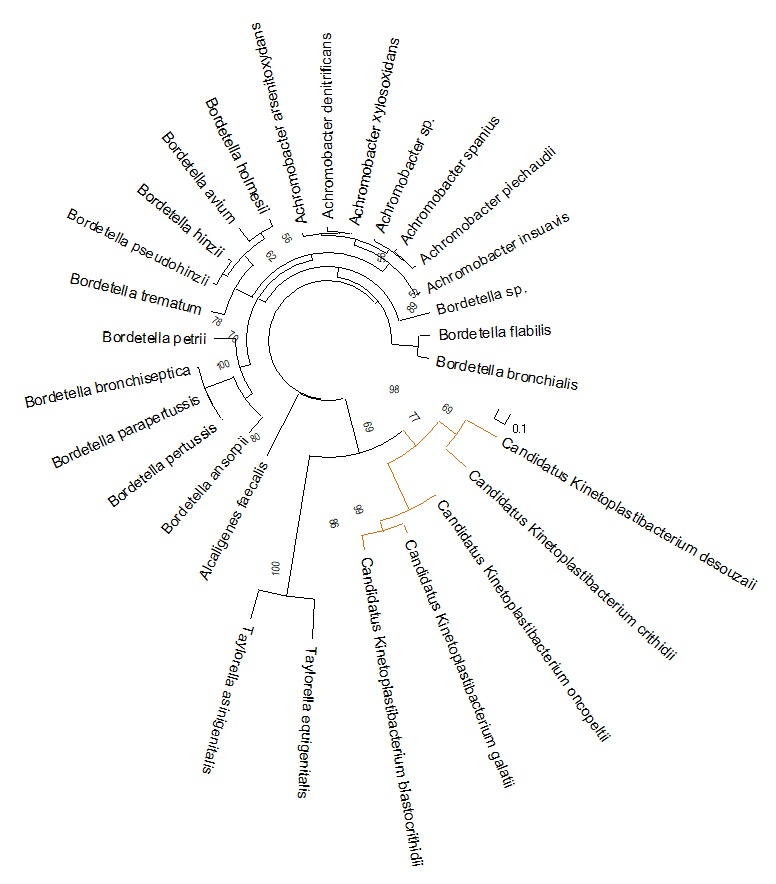

Supplement: Supplementary file 1 [file pathogens-11-00041-s001.zip › SupplFig5.jpg]

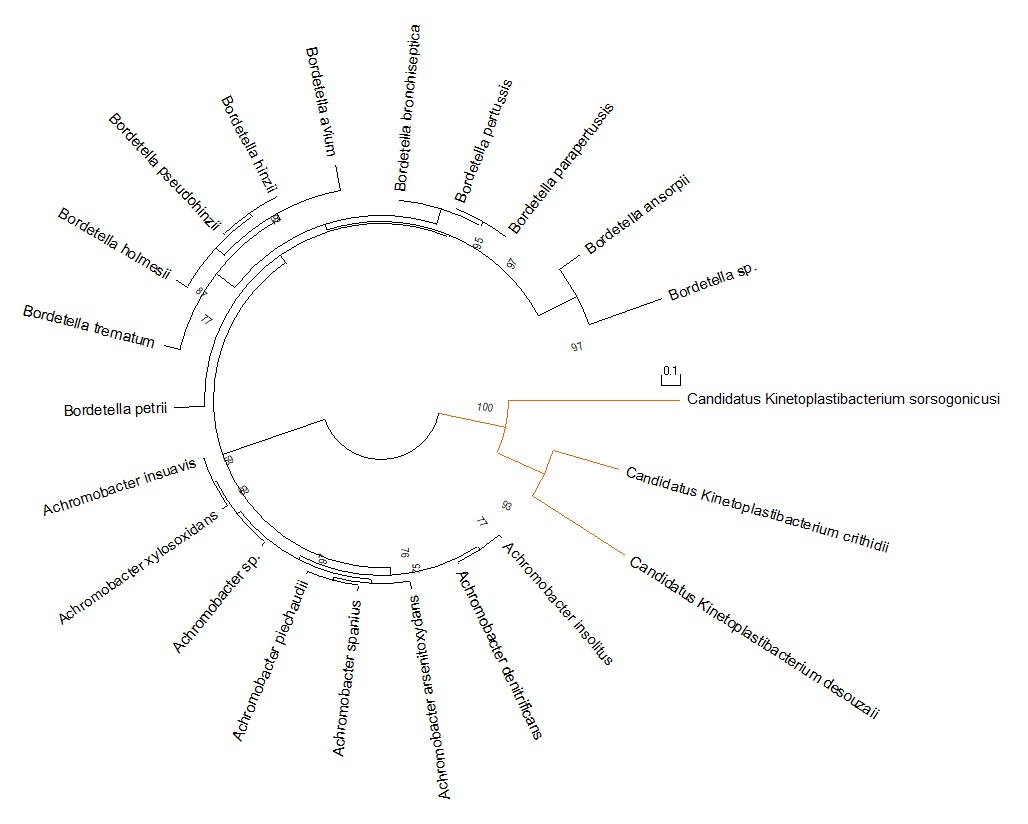

Supplement: Supplementary file 1 [file pathogens-11-00041-s001.zip › SupplFig6.jpg]

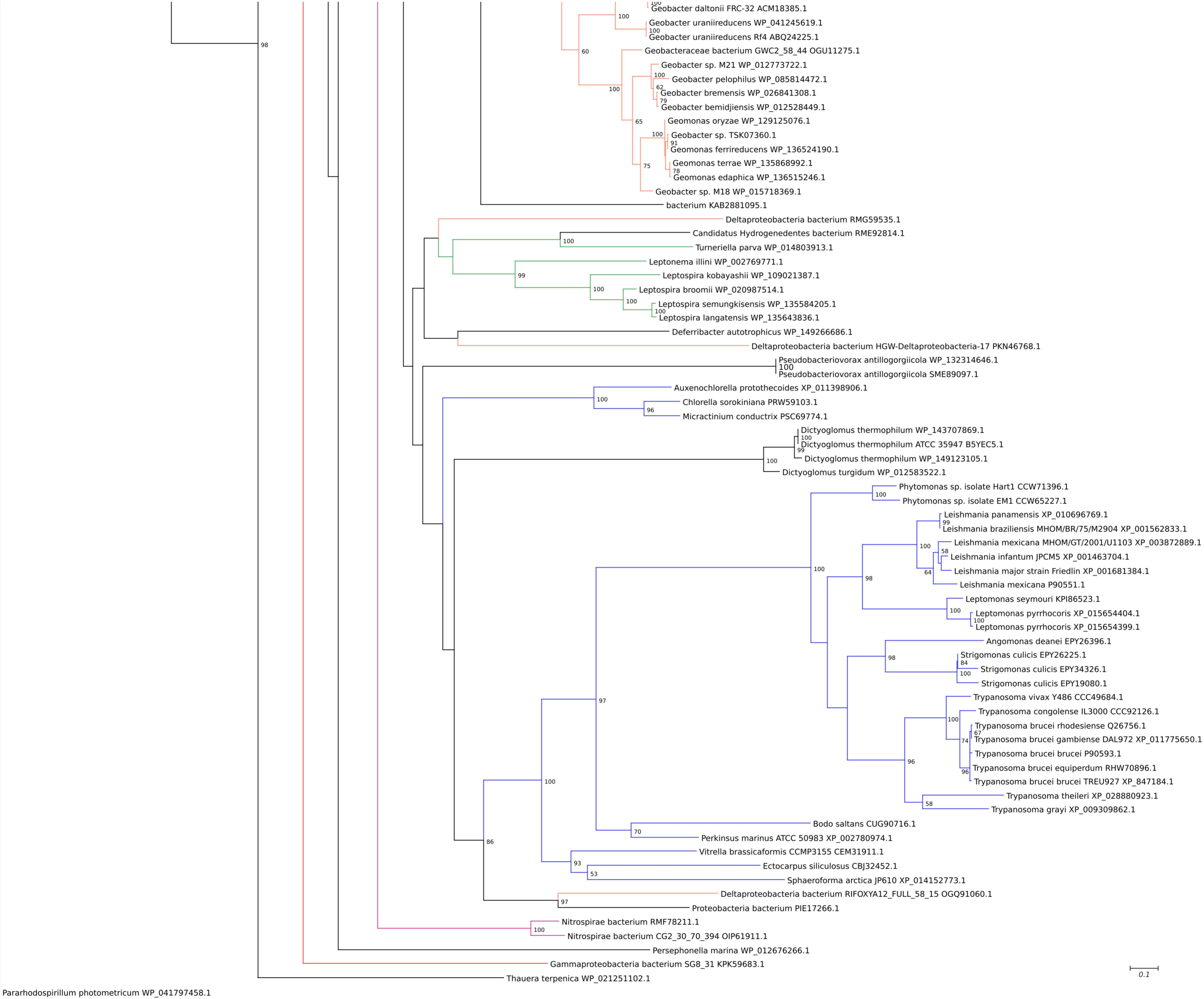

Pararhodospirillum photometricum WP\_041797458.1

Supplement: Supplementary file 1 [file pathogens-11-00041-s001.zip › SupplFig7.pdf]

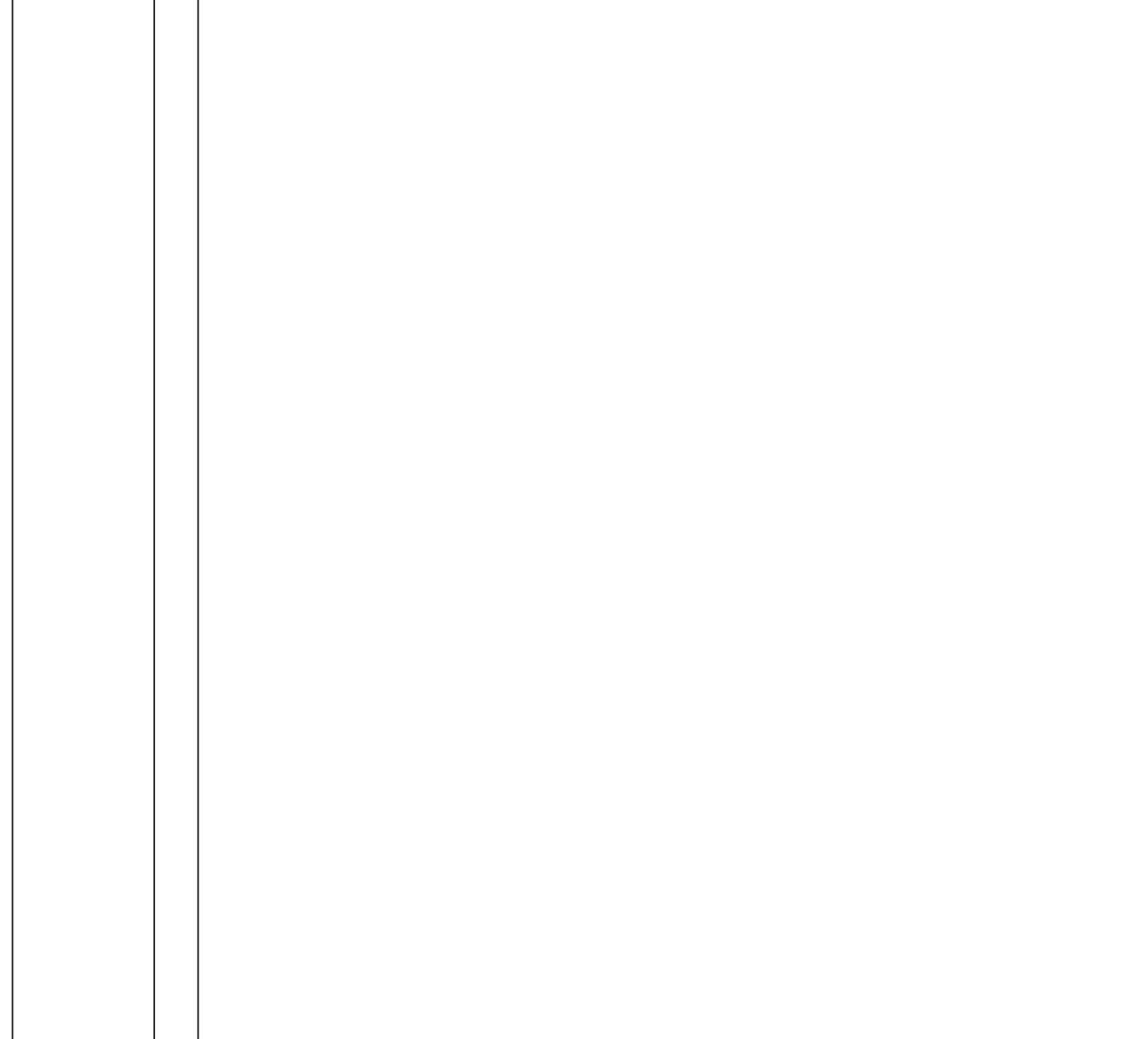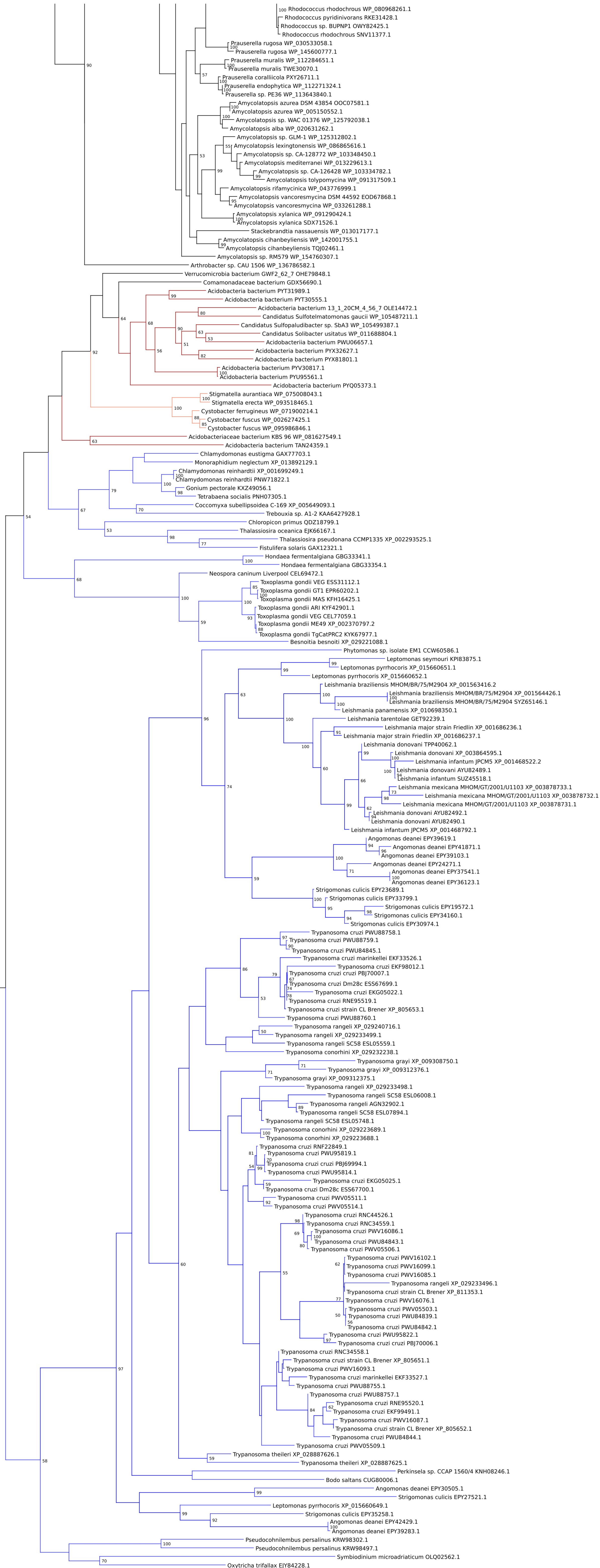

Supplement: Supplementary file 1 [file pathogens-11-00041-s001.zip › SupplFig8.pdf]

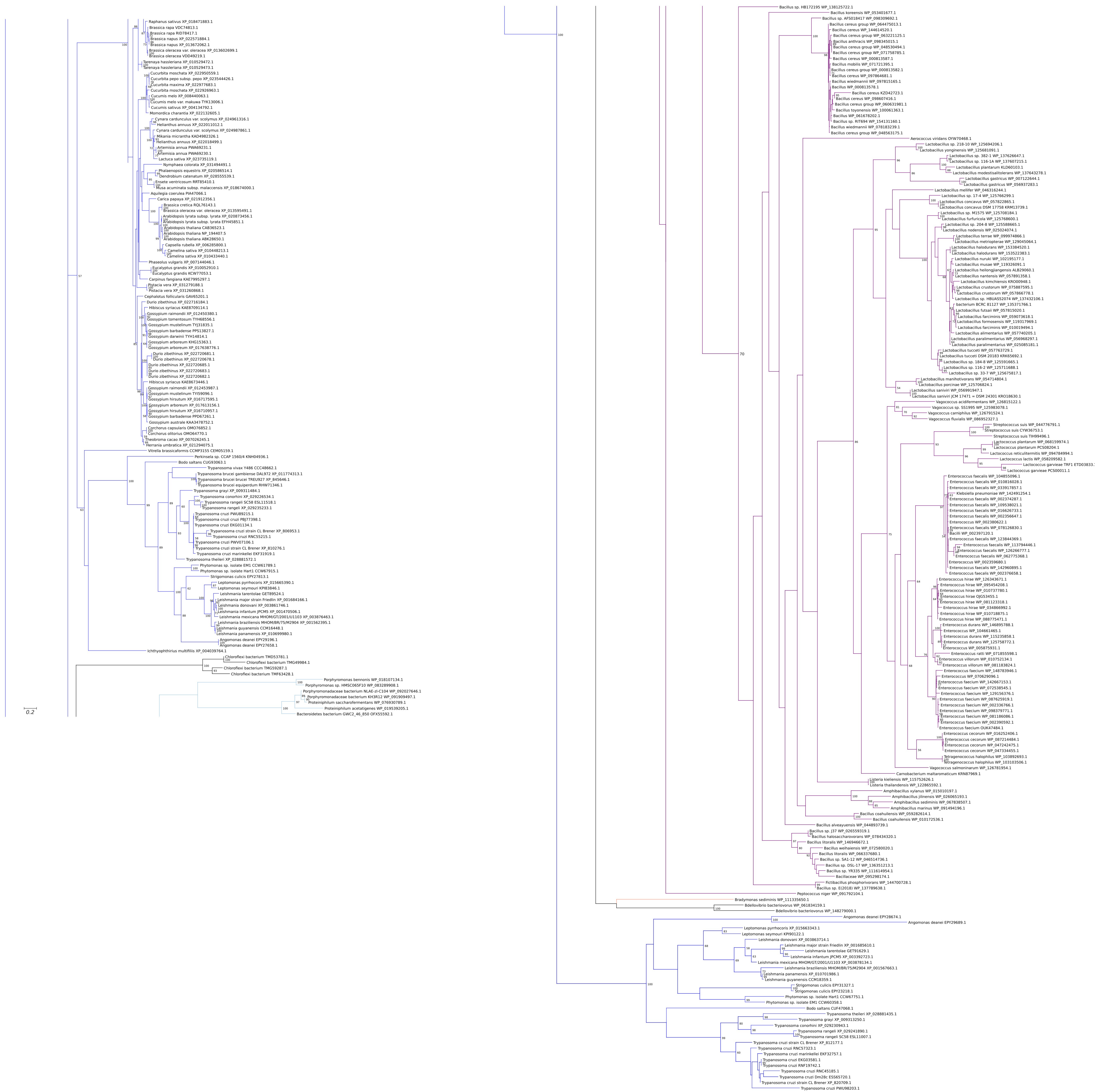

Supplement: Supplementary file 1 [file pathogens-11-00041-s001.zip › SupplFig9.pdf]
